# Nursing intervention to meet the family members’ needs during the surgery waiting time*

**DOI:** 10.1590/1518-8345.5028.3483

**Published:** 2021-10-29

**Authors:** Alejandra Fuentes-Ramirez, Olga Lucia Laverde-Contreras

**Affiliations:** 1 Universidad de La Sabana, Facultad de Enfermería y Rehabilitación, Chía, Cundinamarca, Colombia.

**Keywords:** Perioperative Care, Operating Rooms, Nursing, Family, Clinical Nursing Research, Patient Satisfaction, Cuidados Preoperatorios, Quirófano, Enfermería, Familia, Investigación en Enfermería Clínica, Satisfacción del Paciente, Assistência Perioperatória, Salas Cirúrgicas, Enfermagem, Família, Pesquisa em Enfermagem Clínica, Satisfação do Paciente

## Abstract

**Objective:**

to assess the effect of a care intervention focused on meeting the needs of family members of surgical patients during the surgery waiting time, when compared to conventional care.

**Method:**

a study with a quasi-experimental design that was developed from December 2019 to February 2020 and included 313 family members (Intervention Group=149 and Control Group=164) from a private hospital. The intervention consisted in four moments: “knowing the surgical environment and process”, “information when the surgery starts”, “information when the surgery ends”, and “family-patient reunion”. The “satisfaction” variable was assessed through the “Patient Satisfaction with Nursing Care Quality Questionnaire” instrument. The data were analyzed using descriptive and analytical statistics. The study observed the ethical principles in research.

**Results:**

the family members in the Intervention Group presented greater satisfaction with Nursing care, 90.07(9.8), when compared to the Comparison Group, 78.72(16.38), with an 11.35-point increase(p=0.000).

**Conclusion:**

the results showed that the families that received the intervention on the patient’s status during the surgery waiting time were more satisfied with Nursing care in comparison to the conventional intervention.

## Abstract (es)

**Objetivo:**

evaluar el efecto de una intervención de cuidado centrada en satisfacer las necesidades de los familiares de pacientes intervenidos quirúrgicamente durante la espera quirúrgica en comparación con el cuidado convencional.

**Método:**

diseño cuasi experimental que se desarrolló durante los meses de diciembre de 2019 a febrero de 2020 que incluyó 313 familiares (grupo de intervención = 149 y grupo de control = 164) de un hospital privado. La intervención estuvo compuesta por cuatro momentos: “conocer el ambiente y proceso quirúrgicos”, “información sobre el inicio de la cirugía”, “información sobre elfin de la cirugía”, “reencuentro familia-paciente”. La variable de satisfacción se midió a través del instrumento “Cuestionario satisfacción del paciente con la calidad del cuidado de enfermería”. Los datos fueron analizados con estadística descriptiva y analítica. El estudio tuvo en cuenta los principios éticos en investigación.

**Resultados:**

los familiares del grupo de intervención presentaron una mayor satisfacción con el cuidado de enfermería 90,07 (9,8) comparado con el grupo de comparación 78,72 (16,38), con un aumento de 11,35 puntos (p = 0,000).

**Conclusión:**

los resultados demostraron que las familias que recibieron la intervención sobre el estado del paciente durante el tiempo de espera quirúrgico tuvieron una mayor satisfacción con la calidad del cuidado de enfermería en comparación con la intervención convencional.

## Abstract (pt)

**Objetivo:**

avaliar o efeito de uma intervenção assistencial voltada para o atendimento das necessidades dos familiares de pacientes operados durante a espera no centro cirúrgico em comparação ao atendimento convencional.

**Método:**

estudo quase-experimental desenvolvido entre os meses de dezembro de 2019 e fevereiro de 2020 incluindo 313 familiares (grupo intervenção = 149 e grupo de comparação = 164) de um hospital privado. A intervenção consistiu em quatro momentos: “conhecer o ambiente e o processo cirúrgico”, “informações sobre o início da cirurgia”, “informações sobre o término da cirurgia”, “reunião família-paciente”. A variável de satisfação foi medida por meio do instrumento “Questionário de satisfação do paciente com a qualidade dos cuidados de enfermagem”. Os dados foram analisados com estatística descritiva e analítica. O estudo levou em consideração os princípios éticos em pesquisa.

**Resultados:**

os familiares do grupo intervenção apresentaram maior satisfação com os cuidados de enfermagem 90,07 (9,8) em relação ao grupo de comparação 78,72 (16,38), com aumento de 11,35 pontos (p = 0,000).

**Conclusão:**

os resultados demonstraram que as famílias que receberam a intervenção sobre a condição do paciente durante o tempo de espera cirúrgica relataram maior satisfação com a qualidade dos cuidados de enfermagem se comparados à intervenção convencional.

## Introduction

Surgeries are stressing moments for patients and families due to the death risk implied, as well as to the sequelae after recovery^(1)^; therefore, it is necessary to meet the families’ needs arising in this process.

During the surgery and when the patient enters the operating room, the family loses contact and experiments a series of concerns that are strongly related to the scarce information provided in relation to what will happen in the surgical environment. This lack of information triggers anxiety and fear in the patient’s family members^(2)^.

During the surgery waiting period, the family members must be informed as to whether the procedure is demanding more time than the expected and offered the possibility of communicating with the surgeon after the procedure^(3)^. In turn, the uneasy situation becomes one of the main barriers to learning and, as time progresses^(4)^ and the family remains unaware of what is happening to the patient, these fears are increased, the family members’ need for information is high^(5)^, and many family members can feel dissatisfied with the support and information received^(6)^.

Knowledge about the surgical environment and the patients’ ability to prevent post-operative complications^(7)^ is of great interest for the peri-operative nurses, since it is fundamental for them to carry out innovating strategies in a uniform and structured manner for the patients and their family members^(8)^, as it is them that connect the relatives and enhance satisfaction with care.

It is important to develop educational strategies from Nursing, capable of addressing specific needs of each individual and applying them at the right moment, in order to avoid generic information that saturates and confuses the patients and the care provided to them^(9)^. In this sense, multimedia education in the video format has been increasingly applied, since it is an ideal means which offers the advantage of providing relatively useful information to the patients, as it saves time and is easy to understand^(10)^. In addition, it is advantageous to systematically involve the family members in the patients’ peri-operative care. Nevertheless, the interventions and the involvement degree of the family member in the patient’s care must be adapted to the cultural context^(11)^.

In this sense, the objective of the study was to assess the effect of a care intervention focused on meeting the needs of family members of surgical patients during the surgery waiting time, when compared to conventional care.

## Method

### Design

Quasi-experimental with two groups: experimental group and non-randomized comparison group.

### Population

The research was conducted with family members of patients who were in the surgery waiting room.

### Collection locus

A health institution in Chía, from the Cundinamarca Department, Colombia. The institution has four operating rooms with modern equipment adapted for complex and minimally invasive surgeries(from laparoscopies to microsurgery and laser surgery). The institution specializes in general surgery, orthopedic surgery and thoracic surgery.The patients who access the service come from the neighboring region of the institution and belong to the contributive health regime, to which they are linked by paying a fee, individual and family group, or through a previous funded contribution.

### Time period

During December 2019 and January and February 2020.

### Selection criteria

Selection was for convenience, based on the following pre-requisites: individuals over 18 years old who accompanied their family member during the surgery, remained in the waiting room of the hospital institution during the surgical intervention, and those people for whom the surgeries were scheduled. The family members excluded were those of surgical patients under 18 years old, who were previously hospitalized in specialized care units(intensive, intermediate and coronary), as well as family members with mental disorders.

### Sample

The following algebraic expression was used to calculate the sample size for each group, as well as to determine the required size to ground judgment of the null hypothesis of means equality, assuming homoscedasticity and the condition of balanced sample size^(12)^.

This expression corresponds to:

$$n=2{\left\{\sqrt{\chi_{1-\alpha }^{2}(k-1)-(k-2)}+z_{1-\beta }\right\}}^{2}{\left(\frac{\delta }{\Delta }\right)}^{2}$$

Where: n=The sample size of each group

α=The probability of TypeI error

β=The probability of TypeII error k=The number of groups χ1-α 2 (& - 1)

Percentile 100(1-α) of a chi-square distribution with (k-1)degrees of freedom

*z*1-β: Percentile100(1-β) of a standard normal distribution

σ: The standard deviation of the variable of interest

∆: Maximum difference between means for consideration of β.

Within the design of the samples related to the assessment of the intervention effect, this research assumes the probabilities of TypeI and TypeII errors as α=0.01, β=0.05, since it is necessary to more rigorously control TypeI error, due to the repercussions in the care practice.

Likewise, another precision element for the analysis of the results consists in assuming the delta value as one fourth of the standard deviation(Δ=0.25δ). Consequently, the sample size derived from these elements and assumed for the research is 143 family members in each group(experimental and comparison), that is, 286 family members. However, the authors preferred to exceed the minimum sampling size, reason why a total of 313 family members eventually participated in the research.

The “satisfaction with Nursing care” variable was assessed through the “Patient Satisfaction with Nursing Care Quality Questionnaire”(PSNCQQ) instrument. Reliability (Cronbach’s Alpha:0.94, Content validity:0.9)^(13)^.

Based on the qualitative results of a study that aimed at knowing the experience of the family members of surgical patients during the surgery waiting time^(14)^, the intervention was developed following the guidelines for the development of Nursing interventions^(15)^.

The following were established as modifiable factors: information delivered to the family member about the patient’s surgical process, satisfaction with Nursing care, contact between the Nursing team and the family member, and entrance restriction to the operating rooms.

Non-modifiable factors: patient’s clinical condition and surgical times.

Intervention elements: information about the surgical process during the surgery waiting time and the pre-, intra- and post-operative periods, and family member-patient reunion. Expected results: family member informed during the surgery waiting time, satisfaction with Nursing care, and increased nurse-family member contact. Delivery strategy: individual. Resources: Video, waiting room television, tablet to enter information about the patient’s transfer. Execution environment or locus: waiting room, post-anesthetic recovery room.

Details of the intervention moments: four moments were defined, which are described below, accounting for a total of 20 minutes:

Moment 1, called “knowing the surgical environment and process”. It is based on the presentation of a three-minute long video describing the operating rooms and the pre-surgical preparation, intra-operative and post-operative stages, as well as the information channels available for the family member to know the surgery start and end times on the waiting room television and, later, reunion with the patient at the end of post-anesthetic recovery. The final step is answering the family members’ questions. Time: 6 minutes.

Moment 2, called “information when the surgery starts”. When the patient enters the surgery, it is verified that the Nursing staff loads the information about the surgery start time into the system for sharing information with the family memberand, subsequently, it is verified that the family member has learned about the surgery start time through the waiting room television offering the information. Time: 2 minutes.

Moment 3, called “information when the surgery ends”. When the patient’s surgery ends, it is verified that the Nursing staff has entered the information about the end of the surgery into the system for sharing information with the family member and, subsequently, it is verified that the family member has learned about the end of the surgery through the waiting room television. Time: 2 minutes.

**Figure 1:**
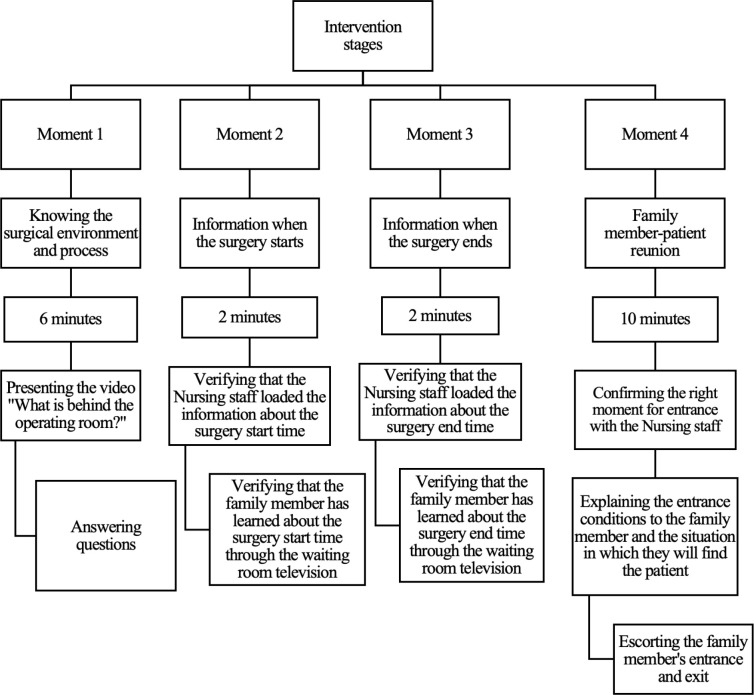
Intervention stages

Moment 4, called “family member-patient reunion”. When the patient ends their post-anesthetic recovery and is in a stable condition after confirming with the Nursing staff the appropriate moment for the family member to enter the post-anesthetic recovery area. The family member is explained the entrance conditions, specifying the situation in which they will find the patient(general status, use of medical devices, need for rest, and limited speaking), as well as the maximum of five minutes for the reunion. The family member’s entrance and exit are escorted.Time: 10 minutes. Figure 1 illustrates the intervention stages.

For data collection, the patients were identified through the institution’s daily surgery schedule; the family members were contacted when the patient entered the surgery, after verifying that the inclusion criteria were met. The patient was contacted, the researcher introduced himself, and the study objectives were explained, as well as the benefits of participating in the research. Likewise, the patient was explained that anonymity would be maintained, that participation was free and voluntary, and that agreement or not to participate would imply no negative consequences in the care provided, either to them or to their relative. The informed consent procedure was conducted in a room aiming to preserve privacy and comfort; the time spent to explain the consent form was approximately 10 minutes. Finally, the process was ended with the participant signing the informed consent document.

The intervention was conducted in its entirety in a personalized manner with each family member. At the end of the intervention, and after the family member-patient reunion, both filled out a printed copy of the Patient Satisfaction with Nursing Care Quality Questionnaire(PSNCQQ)^(13)^.

The comparison group received the institution’s conventional care, consisting in an informative meeting with the physician at the end of the surgery. As compensation, at the end of the assessment, the research team handed the family member a booklet with information about the patient’s home post-operative care; in addition, a space was created to clarify doubts regarding this topic.

### Data analysis

For a general evaluation of the answers obtained, they were consolidated in an Excel database file. The data were analyzed using the Statistical Package for the Social Sciences(SPSS) statistical program, version 24. In the statistical analysis, the categorical variables were expressed through absolute and relative frequencies; and the quantitative variables, through mean and standard deviation. The data normality assumptions were assessed using the Shapiro-Wilk test. The Mann-Whitney Utest was used to compare performance between the groups. The standard significance level of 0.05 for the *p*-value and 95% confidence interval was adopted.

### Ethical aspects

The research was initiated after approval by the Committees of Ethics in Research with Human beings of the Universidad de La Sabana and of the health institution where the research was conducted. The research was carried out considering the ethical precepts required for research with human beings.

## Results

The Comparison Group(CG) consisted of 164 family members, and the Intervention Group(GI) comprised 149 family members. Table 1 shows the sociodemographic data. In the CG, the mean age was 43±14.17 years old, with a minimum of 18 and a maximum of 77; in the IG, the mean was 43±14.25 with a minimum of 18 and a maximum of 78. Most of them were women(65.8%), with complete high school(31.6%), employees(53%), married(48%), their kinship with the surgical patient was that of spouse(36%), having accompanied the family member to the pre-surgical consultation(55%), and having had the experience of accompanying the family member during the surgery waiting time(55%).

**Table 1:** Sociodemographic data of the family members(n=313). Chía, Cundinamarca, Colombia, 2020

Variable	Comparison	%	Intervention	%	Total in the study	%
	N (164)		N (149)		N (313)	
Gender						
Female	110	67.1	96	64.4	206	65.8
Male	54	32.9	53	35.6	107	34.2
Schooling level						
Can read/write	2	1.2	0	0	2	0.6
Elementary School	22	13.4	10	6.7	32	10.2
Bachelor’s Degree	56	34.1	43	28.9	99	31.6
Technician/Technologist	33	20.1	39	26.2	72	23
University	34	20.7	36	24.2	70	22.4
Post-graduate studies	17	10.4	21	14.1	38	12.1
Elementary School	17	10.4	21	14.1	38	12.1
Bachelor’s Degree	87	53	67	45	154	49.2
Occupation						
Unemployed	3	1.8	10	6.7	13	4.2
Household chores	39	23.8	25	16.8	64	20.4
Retiree	8	4.9	10	6.7	18	5.8
Other	27	16.5	37	24.8	64	20.4
Marital status						
Single	36	22	44	29.5	80	25.6
Married	80	48.8	70	47	150	47.9
Consensual union	35	21.3	28	18.8	63	20.1
Separated	7	4.3	6	4	13	4.2
Widowed	6	3.7	1	0.7	7	2.2
Kinship with the surgical patient						
Brother/Sister	9	5.5	14	9.4	23	7.3
Son/Daughter	50	30.5	39	26.2	89	28.4
Father/Mother	35	21.3	25	16.8	60	19.2
Spouse	58	35.4	54	36.2	112	35.8
Other	12	7.3	17	11.4	29	9.3
Accompanied the family member to the pre-surgical consultation						
Yes	96	58.5	77	51.7	173	55.3
No	68	41.5	72	48.3	140	44.7
Has lived the experience of accompanying the family member						
Yes	96	58.5	77	51.7	173	55.3
No	68	41.5	72	48.3	140	44.7

In relation to the study objective of assessing the effect of a care intervention focused on meeting the needs of family members of surgical patients during the surgery waiting time, comparing it with conventional care, it was found that, globally, greater satisfaction of the family members who received the intervention was evident, when compared to the group that did not receive the intervention, with an 11.35-point increase(p=0.000). Table 2 presents the comparison of the “patient satisfaction with Nursing care” dimensions between the comparison and intervention groups.

**Table 2:** Comparison of “patient satisfaction with Nursing Care” between the groups (n=313). Chía, Cundinamarca, Colombia, 2020

	Comparison n=164				Intervention n=149		
	Mean (SD)	Min		Max	Mean (SD)	Min	Max
Concern and care	26.85 (6.4)		7	35	31.09 (3.86)	14	35
Information	23.01 (5.7)		7	30	26.83 (3.4)	12	30
Overall quality	17.01 (2.9)		6	20	18.71 (1.6)	11	20
Care coordination	3.91 (1.1)		1	5	4.50 (0.7)	1	5
Discharge instructions	3.73 (1.1)		1	5	4.42 (0.6)	1	5
Privacy	4.20 (1)		1	5	4.52 (0.6)	1	5
Total Results	78.72 (16.38)		31	100	90.07 (9.8)	44	100

In the «Concern and care» dimension, the IG presented a 4.24-point increase that allows it to rate the care provided as excellent, with significant differences(p=0.000).

In the «Information» dimension, the group that received the intervention considers care quality as excellent, in contrast with the comparison group, which considers it as good(p=0.000).

In the «Overall quality» dimension, it is considered good in both groups. The group that received the intervention did not present significant differences with the comparison group; only a 1.70-point increase was observed(p=0.000).

The questions related to the “Care coordination and discharge instructions” aspect obtained significantly better scores in the intervention group, when compared to the group that did not receive the intervention. They went from being considered as with a good score to presenting excellent scores.

The results suggest that an intervention based on informing the family about what is happening to the patient during the surgery waiting time exerts an influence on the improvement of patient satisfaction with Nursing care quality. In this aspect, the Nursing team plays a fundamental role for its development.

## Discussion

Meeting the family members’ needs during the surgery waiting time must be considered as a quality attribute and, therefore, interventions that improve this process must be developed. According to the results obtained in this research, Nursing concern and care is the dimension with the greatest change after the intervention, a result that is coherent with previous studies which emphasize that the actions performed by the Nursing staff are a priority for the users of the peri-operative area^(16)^.

Likewise, the information about the patient’s situation is acknowledged as an important dimension for the family members. A number of studies show that the family needs to know about the patient’s situation^(17)^; it is for this reason that the interventions that provide information about what the patient will hear, smell, see, taste or feel during the surgical process^(18)^ are valued as positive. In this sense, Nursing care is an effective factor in attaining quality in the health service^(19)^.

In relation to the “Discharge instructions” dimension, the synthesis of the available literature to the present day suggests that the health professionals should focus on educating the patient and on exchanging information to support both patients and caregivers^(20)^. The literature points out that health care during this stage is a topic of interest and that its development must be continued since, although the patients report having received discharge instructions, they lack details and are certainly limited regarding the follow-up actions in case problems arise. More attention is needed to proactively involve the informal caregivers that can facilitate the implementation of discharge plans to improve the patients’ outcomes. Likewise, there is a limited approach in the literature about the study of hospital-home care transition interventions; this is especially relevant since transitional care interventions depend to a large extent on numerous macro-factors, such as the infrastructure and the medical care resources^(21)^. Helping the patients to better know their condition and offering them basic skills to daily manage their diseases can result in physical and psychological benefits for the patient and, in some cases, reduce their dependence on the service^(22)^.

Likewise, the patients’ privacy is fundamental for care, since its absence can be interpreted as lack of humanization in the Nursing service^(23)^. There can be oversimplification of the patient’s perspective regarding this topic and its impact, given the scarcity of research studies in the area^(24)^. The evidence suggests that, although in this study the family members’ perceptions about the care provided were good and the value was high, it is necessary to review the impact of using technologies in health to mitigate the concerns about privacy.

As the family members of surgical patients play an important role in their recovery, their needs must be met during hospitalization and after discharge^(25)^. It is for this reason that it is necessary to continue developing research studies in this area which allow caring for the family member from the moment the surgical process is initiated, during the surgery waiting time and hospitalization, up to the discharge time, by means of a team that can meet the needs arising during this period. As this study was only limited to the surgery waiting time and addressed only one institution, studies with a broader scope are needed which incorporate several institutions at a larger scale.

On the other hand, this research contributes to the advancement of scientific knowledge by describing and testing a Nursing intervention that addresses the family during the surgery waiting time. In this way, compliance with the recommended intervention contributes to the comprehensive care provided to the families that have a surgical patient. These results can stimulate the development of strategies with this population and foster new research studies to support evidence-based practice.

## Conclusion

By means of a care intervention, the research sought to assess to what extent the needs of family members of surgical patients are met during the surgery waiting time. In this sense, the results showed the effectiveness of the intervention in improving satisfaction in the intervention group, in relation to the comparison group. The results emphasize the performance of the Nursing team in the assistance provided to the family members during the surgery.

The development of the Nursing intervention comprised four moments: knowing the surgical environment and process through a video, receiving information when the surgery starts, receiving information when the surgery ends, and favoring the family member-patient reunion. Such intervention proved to be of great relevance, reason why it is necessary to provide continuity in the process of caring for the family in the post-operative period.
